# Thermochromic Luminescent Materials and Multi-Emission Bands in d^10^ Clusters

**DOI:** 10.1038/srep45537

**Published:** 2017-03-30

**Authors:** Romain Gautier, Camille Latouche, Michael Paris, Florian Massuyeau

**Affiliations:** 1 Institut des Matériaux Jean Rouxel (IMN), Université de Nantes, CNRS, 2 rue de la Houssinière, BP 32229, 44322 Nantes Cedex 3, France

## Abstract

Thermochromic luminescent compounds with d^10^ metal ions are interesting materials for applications such as sensors or display devices. However, these properties are difficult to predict prior to their synthesis. In this communication, we investigated materials with structural assemblies known to be responsible of distinct luminescence mechanisms and show that they can be interesting potential thermometers. Thus, we compared the synthesis of a zinc halide and a copper halide based compounds which only differ in their ability to create clusters with metallophilic interactions. The compounds synthesized by hydrothermal method have been structurally characterized by Single-crystal X-ray diffraction, Solid-State NMR, FTIR, UV-Visible spectroscopy, thermal analysis and EPR. The photoluminescence properties of the two materials have been characterized at different temperatures. The copper bromide compound shows luminescence thermochromism in a wide spectrum of colors owing to the formation of clusters generating multi-emission bands while the zinc bromide exhibits a single emission band and no thermochromism.

Materials exhibiting a controlled photoluminescence as a response to temperature have great promise for applications as luminescent thermometers^1^,^2^,^3^,^4^,^5^,^6^. Thus, in the past decade, much effort has been focused on the synthesis of such functional materials. Irrespective of their mechanism, these phenomena can be observed by variations of color or/and intensity of the luminescence. The change in photoluminescence intensity *vs*. temperature is common because of the competition between non-radiative and radiative decays^7^. The color changes in a wide-range is however less common. This modification of color can either originate from the important shift of an emission band or the presence of two or several emission bands whose intensities vary differently with temperature.

Among the thermochromic luminescent materials, the ones built with transition-metal complexes are of particular interest^8^,^9^,^10^,^11^,^12^. The analysis of the ordered molecular assemblies allows understanding and rationalizing the mechanisms that are involved in these phenomena. For example, compounds with d^10^ metals can exhibit luminescence which can be either of ligand centered, charge transfer or metal-centered nature. When these compounds are of cluster-type, the emitting states will depend on the nature of the polynuclear assemblies (depending on the number of transition metals and of the metal-metal interactions). Thus, the bonding interactions between metal cations with a closed-shell configuration have been extensively investigated owing to their roles in some thermochromic properties^13^,^14^,^15^,^16^,^17^.

As the thermochromic behaviour of luminescence can be of different origins, a rational design of materials exhibiting such properties is difficult. In this communication, we show that one strategy is to focus on compounds in which several photoluminescence bands have different origins. Thus, if different photoemissions arising from distinct mechanisms occur in a single-phase, the responses (especially the intensities) to temperature likely differ. The resulting luminescence color will, thus, be changed. As an illustration, we synthesized two d^10^ metal halides (the new compound [C_6_H_16_N_2_]_3_[Cu_4_Br_6_][Cu_2_Br_6_] (**1**) and the previously reported [C_6_H_16_N_2_]ZnBr_4_ (**2**)) with the same ammonium cation and studied their responses with the change of temperature (Fig. 1)^18^.

The d^10^ metal halide compounds are built of anionic [MX_y_]^z−^ inorganic units, or clusters isolated between the templating molecules of 1,4-dimethylpiperazine-1,4-diium. The new copper bromide crystallizes in the noncentrosymmmetric space-group *P2*_*1*_*2*_*1*_*2*_*1*_ and is built from two clusters: a dinuclear [Cu_2_Br_6_]^4−^ and a tetranuclear [Cu_4_Br_6_]^2−^ (Fig. 2). For this compound, the Cu-Br bond lengths range between 2.406(3) Å < *d* < 2.553(3) Å in [Cu_2_Br_6_]^4−^ clusters and 2.358(3) Å < *d* < 2.478(3) Å for [Cu_4_Br_6_]^2−^ clusters. The Cu-Cu distance is 2.686(3) Å in [Cu_2_Br_6_]^4−^ cluster and 2.667(3) Å < *d* < 3.089(3) Å in [Cu_4_Br_6_]^2−^ cluster. The crystal structure determined at 100 K shows that the molecular packing is little affected by temperature. At low temperature, a free water molecule with a large thermal agitation can also be observed. The previously reported zinc bromide crystallizes in the space-group P2_1_/c and is built from tetrahedral [ZnBr_4_]^2−^ units^18^. FTIR, UV-Visible spectroscopy, and thermal analysis were performed on both compounds. To investigate the origin of the large anisotropic displacement parameters on the atoms of the organic molecules, {^1^H}-^13^C CP-MAS Solid-state NMR has been performed for compound 1 (Supporting Information). The EPR spectroscopy confirmed the absence of Cu(II) in the copper bromide sample. Ground State (GS) and triplet Excited State (ES) computations have been performed on the [Cu_4_Br_6_]^2−^ moiety (computations details are provided in Supporting Information). The optimized GS geometry enforcing the *T*_*d*_ symmetry is in good agreement with experimental data (Cu-Cu = 2.749 Å; Cu-Br = 2.456 Å). In this geometry, each Cu atom is in a trigonal planar configuration bonded to three Br atoms. On the other hand, the halides are linked to two metals.

Experimentally, the absorption spectrum exhibits a very sharp and intense peak around 370 nm (Supporting Information). This information is well reproduced in the calculations since the absorption has been computed using TD-DFT at 366 nm (f = 0.06, *T*_*2*_). Two electronic transitions are computed around 300 nm (305 nm, f = 0.02, *T*_*2*_; 297 nm, f = 0.03, *T*_*2*_), constituting the second band on Figure S1. Finally, an intense peak around 260 nm is constituted of multiple transitions, together with a very intense one computed at 264 nm (f = 0.07).

The steady-state photoluminescence properties of the two compounds have been characterized at different temperatures (Fig. 3). Mapping of the excitation vs. emission allowed identifying the different photoemissions and following their evolutions with temperature. At 77 K, compound **1** shows two emission bands at high energy (about 500 nm) and lower energy (about 700 nm) (Fig. 3(a)). The relative ratio of these two emissions differ for different temperatures. The high energy emission disappears at room temperature. A blueshift of the low energy emission is also observed in function of the temperature. Its maximum is located at 650 nm at 300 K. The luminescence properties of copper halide clusters have been extensively studied. The low energy emission is well-known for cluster Cu_4_ and has been previously attributed to be of cluster centered nature with a combination of halide to metal charge transfer and copper-centered transition^13^. For the zinc bromide, a weak photoemission at 620 nm was observed at low temperature (Fig. 3(b)) but this compound do not show any photoluminescence properties at 300 K.

Time-resolved photoluminescence has been performed on compound **1** (Fig. 4) in order to confirm the presence of triplet excited states (ES) prior to their computation. Figure 4(a) shows the luminescence on the whole visible area. We clearly observed a strong luminescence centered at about 620 nm in the 100 μs time-range and a second band at 490 nm. Photoluminescence decays for the two bands have been averaged based on the red square and on the green square, respectively. The lifetime of the low energy and the high energy emissions are 21 μs and 0.28 μs, respectively. These lifetimes were obtained by fitting the experimental data with a monoexponential decay convoluted with the laser pulse. As in steady-state PL, the time-resolved PL spectrum integrated over the entire 200 μs window at 77 K (Figure S7) exhibits an intense emission band located at 497 nm compared to the intensity of the band located at 657 nm. As in 300 K, the PL decays monoexponentially for the two bands but their lifetimes increase (47.5 μs and 37.7 μs for the bands at 497 nm and 657 nm respectively). This trend, with comparable lifetime values, has been well observed in litterature^19^, and one key to understand this behaviour is probably the temperature-induced rigidity of those systems^20^.

Optimizations of the ES on the Cu_4_ clusters have been performed without symmetry constraint and have been checked to be true minima on the potential energy surface. As a matter of fact they allow an investigation of the origin of the two photoemission bands. Indeed, the computed emission wavelengths (phosphorescence) were around 490 nm and 850 nm. The computed one of lowest energy slightly overestimates the experimental one whereas the second one fits with the observed photoemission. For the lowest energy triplet ES, the metallic tetrahedron is opened and one Cu-Cu interaction is weaken (3.91 Å). One Br atom becomes singly bonded to a Cu with a short Cu-Br distance (2.38 Å). The other Cu-Cu distances are also dramatically affected with a strong shortening from GS to ES (≈10%, 2.51–2.66 Å). In the second ES, the tetrahedron is maintained with six short metal-metal contacts (2.51 to 2.73 Å). In order to assess the strength of the interaction between copper atoms, a coupled NAO (Natural Atomic Orbital) and Wiberg indices analysis has been performed^21^. As a matter of fact, it appears that the “closed” cluster possesses all its Cu-Cu Wiberg bond indices ranging in the same order of magnitude (0.08–0.18). However, in the case of the “opened” cluster, there is one Cu-Cu Wiberg bond index which is one order of magnitude smaller than the others (0.009). This result allows us to confirm that for the opened cluster the d^10^-d^10^ interaction is weak^10^,^11^,^22^,^23^,^24^,^25^,^26^.

Owing to the competition between non-radiative and radiative decays, most luminescent materials show a significant change in the intensity of photoluminescence *vs*. temperature variation. Thus, our two compounds show a decrease of intensity of the photoemission bands when the temperature increases. This phenomenon is of interest for the materials showing several emission bands originating from different mechanisms because the evolution of intensity with temperature for each of these emission bands is more likely to be different. It results that the mixing of the emission bands and, thus, the resulting colour of the photoluminescence will change gradually with temperature. This thermochromic behaviour of the photoluminescence will also be more common in the materials for which distinct mechanisms of the photoluminescence are considered. Thus, one could focus on materials in which the different categories of mechanisms (such as ligand-centered, or metal-centered) are possible. The appropriate choice of ligands can favor ligand-centered or ligand-metal charge transfer mechanism. The selection of metals favoring the formation of different clusters by metallophilic interactions can also be a key to obtain materials with multi-band emissions. Indeed, in our compounds, only the inorganic component is different (absence and presence of polynuclear assemblies for Zn and Cu halides, respectively).

In summary, mechanisms of photoluminescence in materials built from transition metal complexes are usually difficult to identify or predict prior to the synthesis. However, the crystal structures built from assemblies exhibiting multi-emission bands are more likely to exhibit thermochromic behaviour of the photoluminescence. Thus, the copper halide material built from polynuclear assemblies exhibits multi-emission bands that evolve differently with temperature. It also results a thermochromic photoluminescence from 77 K to room temperature in a wide spectrum of colors.

## Methods

### Synthesis

The materials have been hydrothermally synthesized using mixtures of metals (Cu (Merck, 99.7%), Zn (Merck, 99.9%)) and acid (HBr (Alfa Aesar, 48%)). The compounds are synthesized by hydrothermal method (heating at 180 °C during 24 h and slowly cooled down to room temperature at the rate of 10 °C/h) using a 23 mL Teflon-lined stainless steel autoclave. Single crystals suitable for X-ray diffraction were recovered by filtration. CCDC 1482035 and CCDC 1532971 contain the supplementary crystallographic data at room temperature and 100 K, respectively. These data are provided free of charge by The Cambridge Crystallographic Data Centre. Copper bromide (**1**) [C_6_H_16_N_2_]_3_[Cu_4_Br_6_][Cu_2_Br_6_] was synthesized from a mixture of 7.87 mmol of Cu metal, 8.86 mmol of N,N’-dimethylpiperazine and 3 ml of HBr 48%. Zinc bromide (**2**) [C_6_H_16_N_2_]ZnBr_4_ was synthesized from a mixture of 7.65 mmol of Zn metal, 2.95 mmol of N,N’-dimethylpiperazine and 3 ml of HBr 48%.

## Additional Information

**How to cite this article**: Gautier, R. *et al*. Thermochromic Luminescent Materials and Multi-Emission Bands in d^10^ Clusters. *Sci. Rep.* **7**, 45537; doi: 10.1038/srep45537 (2017).

**Publisher's note:** Springer Nature remains neutral with regard to jurisdictional claims in published maps and institutional affiliations.

## Supplementary Material

Supplementary Information

## Figures and Tables

**Figure 1:**
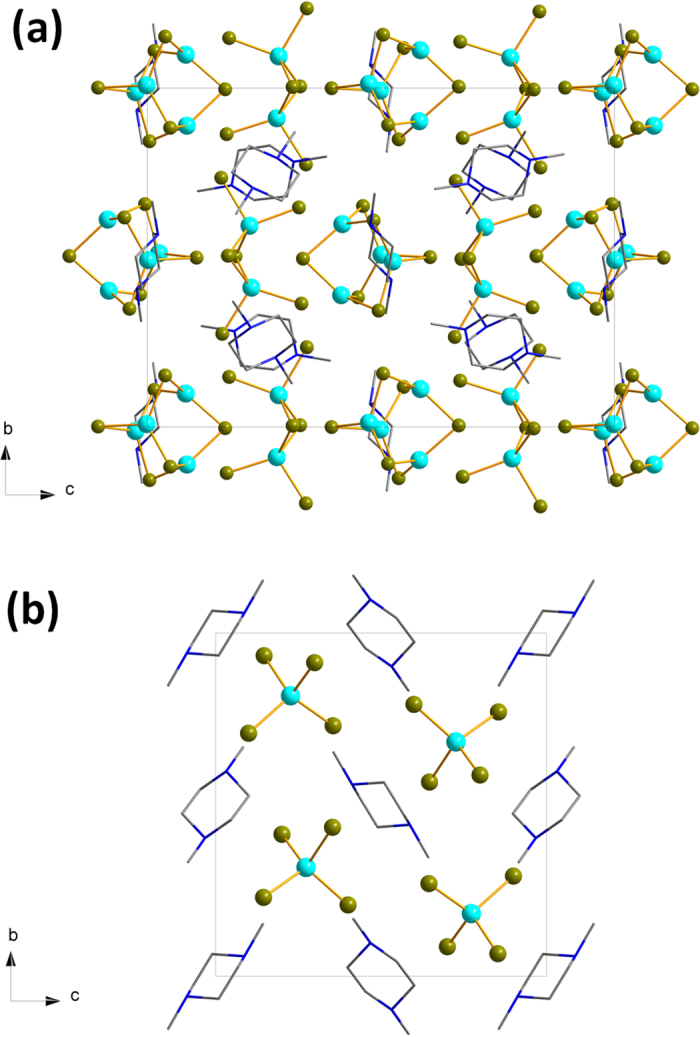
Representation of the two d^10^ metal halides (**a**) [C_6_H_16_N_2_]_3_[Cu_4_Br_6_][Cu_2_Br_6_] (**1**) and (**b**) [C_6_H_16_N_2_]ZnBr_4_ (**2**)).

**Figure 2:**
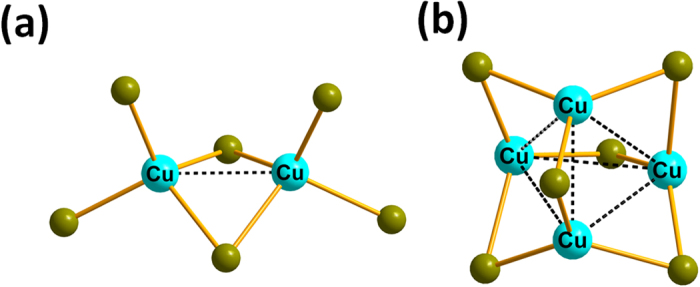
Representation of the copper bromide structures: (**a**) View of the copper dinuclear [Cu_2_Br_6_]^4−^ and, (**b**) the copper tetranuclear [Cu_4_Br_6_]^2−^.

**Figure 3:**
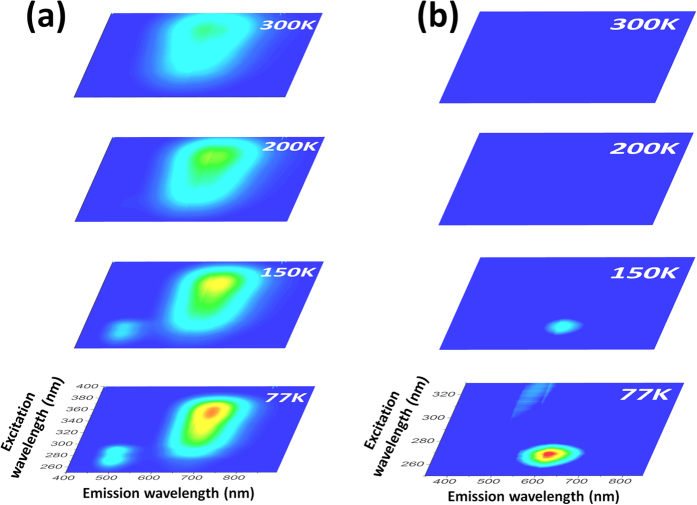
Solid-state photoluminescence (excitation vs. emission) for (**a**) compound **1** and (**b**) compound **2** in function of temperature.

**Figure 4:**
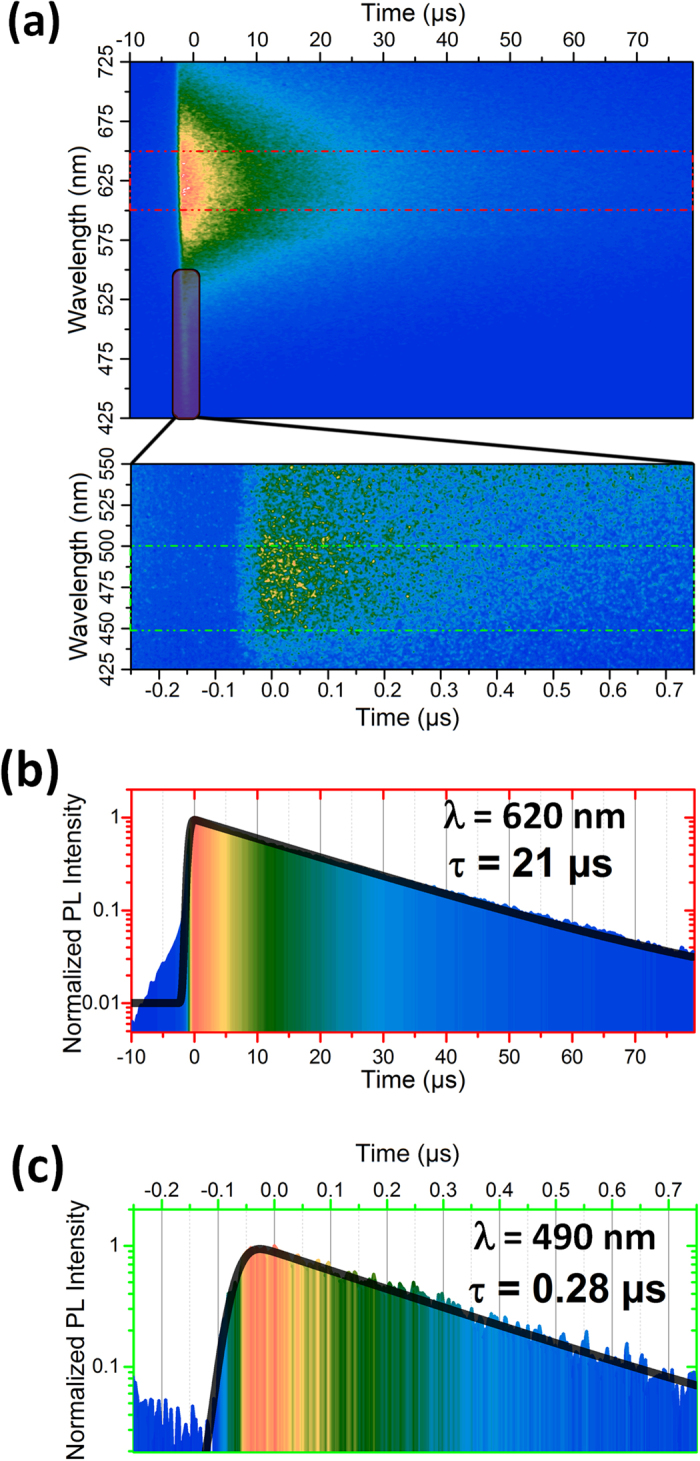
Time-resolved photoluminescence on compound 1 at λ_exc_ = 267 nm: (**a**) Overview of the emission *vs*. decay time in the visible spectrum, (**b**) Decay of the emission band at 620 nm, and (**c**) Decay of emission band at 490 nm.

## References

[b1] Beck J. B. & Rowan S. J. Multistimuli, Multiresponsive Metallo-Supramolecular Polymers. J. Am. Chem. Soc. 125, 13922–13923 (2003). 14611204 10.1021/ja038521k

[b2] Uchiyama S., Prasanna de Silva A. & Iwai K. Luminescent Molecular Thermometers. J. Chem. Educ. 83, 720 (2006).

[b3] Brites C. D. S. . A Luminescent Molecular Thermometer for Long-Term Absolute Temperature Measurements at the Nanoscale. Adv. Mater. 22, 4499–4504 (2010). 20803765 10.1002/adma.201001780

[b4] Shen Y. . Degradable Dual pH- and Temperature-Responsive Photoluminescent Dendrimers. Chem. – Eur. J. 17, 5319–5326 (2011). 21465587 10.1002/chem.201003495

[b5] Cui Y. . A Luminescent Mixed-Lanthanide Metal–Organic Framework Thermometer. J. Am. Chem. Soc. 134, 3979–3982 (2012). 22352469 10.1021/ja2108036

[b6] Rao X. . A Highly Sensitive Mixed Lanthanide Metal–Organic Framework Self-Calibrated Luminescent Thermometer. J. Am. Chem. Soc. 135, 15559–15564 (2013). 24063306 10.1021/ja407219k

[b7] Chen X., Zhang X. & Zhang G. Wide-range thermochromic luminescence of organoboronium complexes. Chem. Commun. 51, 161–163 (2015). 10.1039/c4cc08289c 25387801

[b8] Forward J. M., Assefa Z. & Fackler J. P. Photoluminescence of gold(I) phosphine complexes in aqueous solution. J. Am. Chem. Soc. 117, 9103–9104 (1995).

[b9] Barbieri A., Accorsi G. & Armaroli N. Luminescent complexes beyond the platinum group: the d10 avenue. Chem. Commun. 2185–2193, doi: 10.1039/B716650H (2008). 18463736

[b10] Latouche C. . Shape Modulation of Octanuclear Cu(I) or Ag(I) Dichalcogeno Template Clusters with Respect to the Nature of their Encapsulated Anions: A Combined Theoretical and Experimental Investigation. Inorg. Chem. 52, 7752–7765 (2013). 23750811 10.1021/ic400959a

[b11] Latouche C., Liu C. W. & Saillard J.-Y. Encapsulating Hydrides and Main-Group Anions in d10-Metal Clusters Stabilized by 1,1-Dichalcogeno Ligands. J. Clust. Sci. 25, 147–171 (2013).

[b12] Benito Q. . Geometry Flexibility of Copper Iodide Clusters: Variability in Luminescence Thermochromism. Inorg. Chem. 54, 4483–4494 (2015). 25857746 10.1021/acs.inorgchem.5b00321

[b13] Perruchas S. . Mechanochromic and Thermochromic Luminescence of a Copper Iodide Cluster. J. Am. Chem. Soc. 132, 10967–10969 (2010). 20698644 10.1021/ja103431d

[b14] Benito Q. . Polymorphic Copper Iodide Clusters: Insights into the Mechanochromic Luminescence Properties. J. Am. Chem. Soc. 136, 11311–11320 (2014). 25076411 10.1021/ja500247b

[b15] Benito Q. . Mechanochromic Luminescence of Copper Iodide Clusters. Chem. – Eur. J. 21, 5892–5897 (2015). 25755012 10.1002/chem.201500251

[b16] Yao R.-X., Hailili R., Cui X., Wang L. & Zhang X.-M. A perfectly aligned 63 helical tubular cuprous bromide single crystal for selective photo-catalysis, luminescence and sensing of nitro-explosives. Dalton Trans. Camb. Engl. 2003 44, 3410–3416 (2015). 10.1039/c4dt03657c 25601196

[b17] Fu Z. . Cuprous Iodide Pseudopolymorphs Based on Imidazole Ligand and Their Luminescence Thermochromism. Cryst. Growth Des. 16, 2322–2327 (2016).

[b18] Feist M., Trojanov S. & Kemnitz E. Die Kristallstrukturen von 1,4-Dimethylpiperazinium-tetrabromocobaltat(II) und -zinkat(II), (dmpipzH2) [MIIBr4] (M = Co, Zn)/The Crystal Structures of 1,4-Dimethylpiperazinium Tetrabromocobaltate(II) and -Zincate(II), (dmpipzH2) [MIIBr4] (M = Co, Zn). Z. Für Naturforschung B 51, 9–13 (2014).

[b19] Ford P. C., Cariati E. & Bourassa J. Photoluminescence Properties of Multinuclear Copper(I) Compounds. Chem. Rev. 99, 3625–3648 (1999). 11849032 10.1021/cr960109i

[b20] Khatri N. M. . Luminescence and Nonlinear Optical Properties in Copper(I) Halide Extended Networks. Inorg. Chem. 55, 11408–11417 (2016). 27735188 10.1021/acs.inorgchem.6b01879

[b21] Glendening E. D. *et al. NBO 5.0 Program* (2001).

[b22] Liu C. W. . [Ag7(H){E2P(OR)2}6] (E = Se, S): Precursors for the Fabrication of Silver Nanoparticles. Inorg. Chem. 52, 2070–2077 (2013). 23383855 10.1021/ic302482p

[b23] Edwards A. J. . Chinese Puzzle Molecule: A 15 Hydride, 28 Copper Atom Nanoball. Angew. Chem. Int. Ed. 53, 7214–7218 (2014). 10.1002/anie.201403324 24803070

[b24] Dhayal R. S. . [Ag21{S2P(OiPr)2}12]+: An Eight-Electron Superatom. Angew. Chem. Int. Ed. 54, 3702–3706 (2015). 10.1002/anie.201410332 25631754

[b25] Dhayal R. S. . Diselenophosphate-Induced Conversion of an Achiral [Cu20H11{S2P(OiPr)2}9] into a Chiral [Cu20H11{Se2P(OiPr)2}9] Polyhydrido Nanocluster. Angew. Chem. Int. Ed. 54, 13604–13608 (2015). 10.1002/anie.201506736 26387572

[b26] Dhayal R. S., van Zyl W. E. & Liu C. W. Polyhydrido Copper Clusters: Synthetic Advances, Structural Diversity, and Nanocluster-to-Nanoparticle Conversion. Acc. Chem. Res. 49, 86–95 (2016). 26696469 10.1021/acs.accounts.5b00375

