# Supplementary material for: Thermochromic Luminescent Materials and Multi-Emission Bands in d10 Clusters
Source: Sci Rep. 2017 Mar 30;7:45537. doi: 10.1038/srep45537 (PMC5371983; doi:10.1038/srep45537)
Supplement: Supplementary Information [file srep45537-s1.pdf]

# SUPPORTING INFORMATION

# Thermochromic Luminescent Materials and Multi-Emission Bands in $d^{10}$ Clusters

*Romain Gautier[a,*], Camille Latouche[a,*], Michael Paris[a], Florian Massuyeau[a]*

*Structure determination*

The structure determination of compound **1** was carried out from Single-crystal X-ray diffraction using a Bruker-Nonius Kappa CCD diffractometer with monochromated Mo Kα radiation. Absorption corrections were carried out with SADABS.[1] The crystal structure was determined by direct methods and were completed by Fourier difference syntheses with SIR2004.[2] SHELXL-2013 was used to refine the crystal structures and anisotropic displacement parameters were considered.[3] The CIF was compiled with Olex2.12,[4] and additional symmetry elements were checked with the program PLATON.[5] A summary of the crystallographic data for compound **1** is represented in Table 1.

**Table 1.** Crystallographic data for compound **1**

| | **1** |
|---|---|
| Space group | $P2_12_12_1$ |
| a/Å | 13.6602(9) |
| b/Å | 15.1603(15) |
| c/Å | 20.8040(15) |
| α/° | 90 |
| β/° | 90 |
| γ/° | 90 |
| Radiation | Mo Kα |
| 2Θ range for data collection/° | 12.86 to 51.02 |
| Reflections collected | 27989 |
| Data/restraints/parameters | 7645/0/378 |
| Goodness-of-fit on $F^2$ | 1.060 |
| Final R indexes [I>=2σ (I)] | $R_1$ = 0.0547<br>$wR_2$ = 0.1223 |
| Largest diff. peak/hole / e $Å^{-3}$ | 3.08/-1.92 |

*FT-IR Spectroscopy*

A Bruker Vertex 70 Instrument was used to collect the FT-IR spectra of compounds **1-2**. For each sample, 100 scans were collected from 400 $cm^{-1}$ to 4000 $cm^{-1}$ with a resolution of 4 $cm^{-1}$ (a background spectrum was subtracted). The compounds were mixed with KBr and pressed into pellets.

*UV/Visible Spectroscopy*

A Varian Cary 5G spectrophotometer using a 110 mm integrating sphere was used to collect the diffuse reflectance spectra from 250 nm to 2500 nm. Absorbance spectra were calculated from reflectance measurements using the Kubelka Munk function ($a/S = (1-R)^2/2R$ where $a$ is the absorption coefficient, $S$ the scattering coefficient and $R$ the reflectance).

*Photoluminescence*

Photoluminescence (PL) excitation versus emission maps are obtained on a Horiba Jobin –Yvon Fluorolog-3 equipped with a CCD camera, a 450W Xenon lamp. All measurements were achieved in an Oxford cryostat which allowed (PL) measurements decreasing temperature down to 77K.

Time-resolved photoluminescence (TRPL) experiments were carried out at room temperature with a regenerative amplified femtosecond Ti:Sapphire laser system (Spectra Physics Hurricane X) frequency-tripled to obtain an excitation line $\lambda_{exc}$=267 nm. The transient signals were

spectrally dispersed into a Princeton Instruments SP2300 imaging Acton spectrograph and temporally resolved with a high dynamic range Hamamatsu C7700 streak camera.

*Calculations*

Ground State (GS) and triplet Excited State (ES) computations have been performed on the $[Cu_4Br_6]^{2-}$ moiety using the PBE0 functional,[6,7] together with the Def2TZVPD basis set,[8] using the Gaussian package.[9] All computations have been performed in vacuum and the geometries have been checked to be on the minimum of the Potential Energy Surface (PES) by diagonalizing their Hessian, even for the ES. Simulated electronic absorption spectrum together with oscillator strengths has been modeled using VMS.[10]

*Solid-state NMR*

$\{^{1}H\}$-$^{13}C$ CP-MAS (Cross-Polarization and Magic Angle Spinning) NMR spectra were acquired on Bruker 500 MHz and Bruker 300 MHz Avance III spectrometers using 4 mm CP-MAS probes. In both cases, we used a MAS frequency of 10 kHz, a contact time of 1.5 ms and a $^{1}H$ recycle time of 3s. Spectra were referenced to TMS using the 29.5 ppm resonance of adamantane.

The resolution of the $\{^{1}H\}$-$^{13}C$ CP-MAS NMR spectrum is hardly improved by the increase of the magnetic field from 300 to 500 MHz (7 T to 11.7 T). Therefore, line widths are dominated by isotropic chemical shift distributions reflecting small variation in the $^{13}C$ local geometries. This gives confidence in the accuracy of the proposed crystallographic structure although reported U factors for C atoms are quite high.

REFERENCES


(1) Sheldrick, G. M. *SADABS*; University of Göttingen: Germany, 2002.
(2) Burla, M. C.; Caliandro, R.; Camalli, M.; Carrozzini, B.; Cascarano, G. L.; De Caro, L.; Giacovazzo, C.; Polidori, G.; Spagna, R. *J. Appl. Crystallogr.* **2005**, *38* (2), 381–388.
(3) Sheldrick, G. M. *Acta Crystallogr. Sect. C Struct. Chem.* **2015**, *71* (1), 3–8.
(4) Dolomanov, O. V.; Bourhis, L. J.; Gildea, R. J.; Howard, J. A. K.; Puschmann, H. *J. Appl. Crystallogr.* **2009**, *42* (2), 339–341.
(5) Spek, A. L. *PLATON ; Utrecht University: Utrecht, The Netherlands, 2001.*
(6) Ernzerhof, M.; Scuseria, G. E. *J. Chem. Phys.* **1999**, *110* (11), 5029–5036.
(7) Adamo, C.; Barone, V. *J. Chem. Phys.* **1999**, *110* (13), 6158–6170.
(8) Weigend, F.; Ahlrichs, R. *Phys. Chem. Chem. Phys.* **2005**, *7* (18), 3297–3305.
(9) Frisch, M.; Trucks, G.; Schlegel, H.; Scuseria, G.; Robb, M.; Cheeseman, J.; Montgomery, J.; Vreven, T.; Kudin, K.; Burant, J.; Millam, J.; Iyengar, S.; Tomasi, J.; Barone, V.; Mennucci, B.; Cossi, M.; Scalmani, G.; Rega, N.; Petersson, G.; Nakatsuji, H.; Hada, M.; Ehara, M.; Toyota, K.; Fukuda, R.; Hasegawa, J.; Ishida, M.; Nakajima, T.; Honda, Y.; Kitao, O.; Nakai, H.; Klene, M.; Li, X.; Knox, J.; Hratchian, H.; Cross, J.; Bakken, V.; Adamo, C.; Jaramillo, J.; Gomperts, R.; Stratmann, R.; Yazyev, O.; Austin, A.; Cammi, R.; Pomelli, C.; Ochterski, J.; Ayala, P.; Morokuma, K.; Voth, G.; Salvador, P.; Dannenberg, J.; Zakrzewski, V.; Dapprich, S.; Daniels, A.; Strain, M.; Farkas, O.; Malick, D.; Rabuck, A.; Raghavachari, K.; Foresman, J.; Ortiz, J.; Cui, Q.; Baboul, A.; Clifford, S.; Cioslowski, J.; Stefanov, B.; Liu, G.; Liashenko, A.; Piskorz, P.; Komaromi, I.; Martin, R.; Fox, D.; Keith, T.; Laham, A.; Peng, C.; Nanayakkara, A.; Challacombe, M.; Gill, P.; Johnson, B.; Chen, W.; Wong, M.; Gonzalez, C.; Pople, J. .
(10) Licari, D.; Baiardi, A.; Biczysko, M.; Egidi, F.; Latouche, C.; Barone, V. *J. Comput. Chem.* **2015**, *36* (5), 321–334.

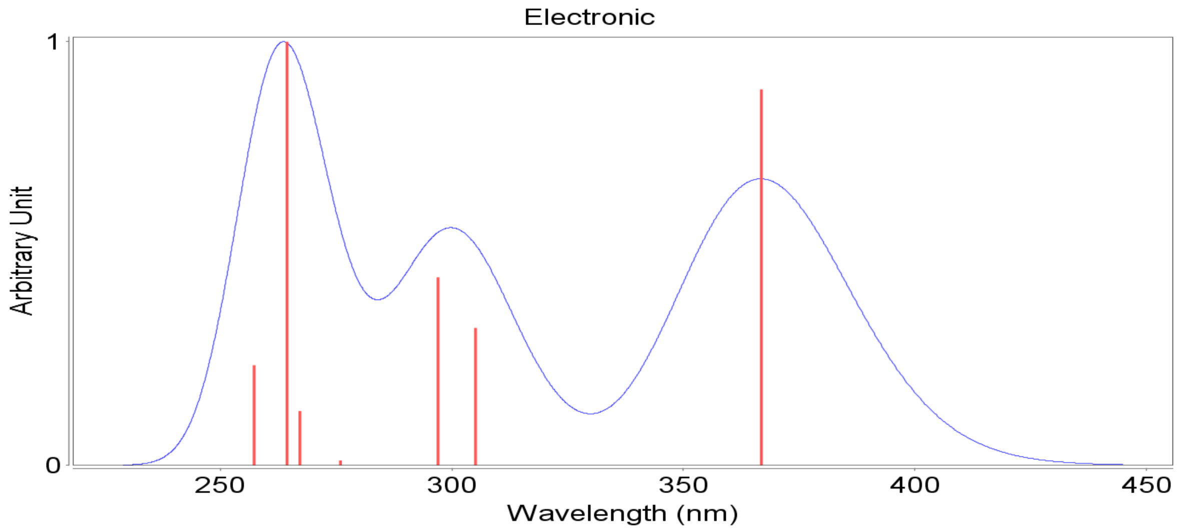


**Figure S1.** Simulated absorption spectrum of the $[Cu_4Br_6]^{2-}$ cluster.

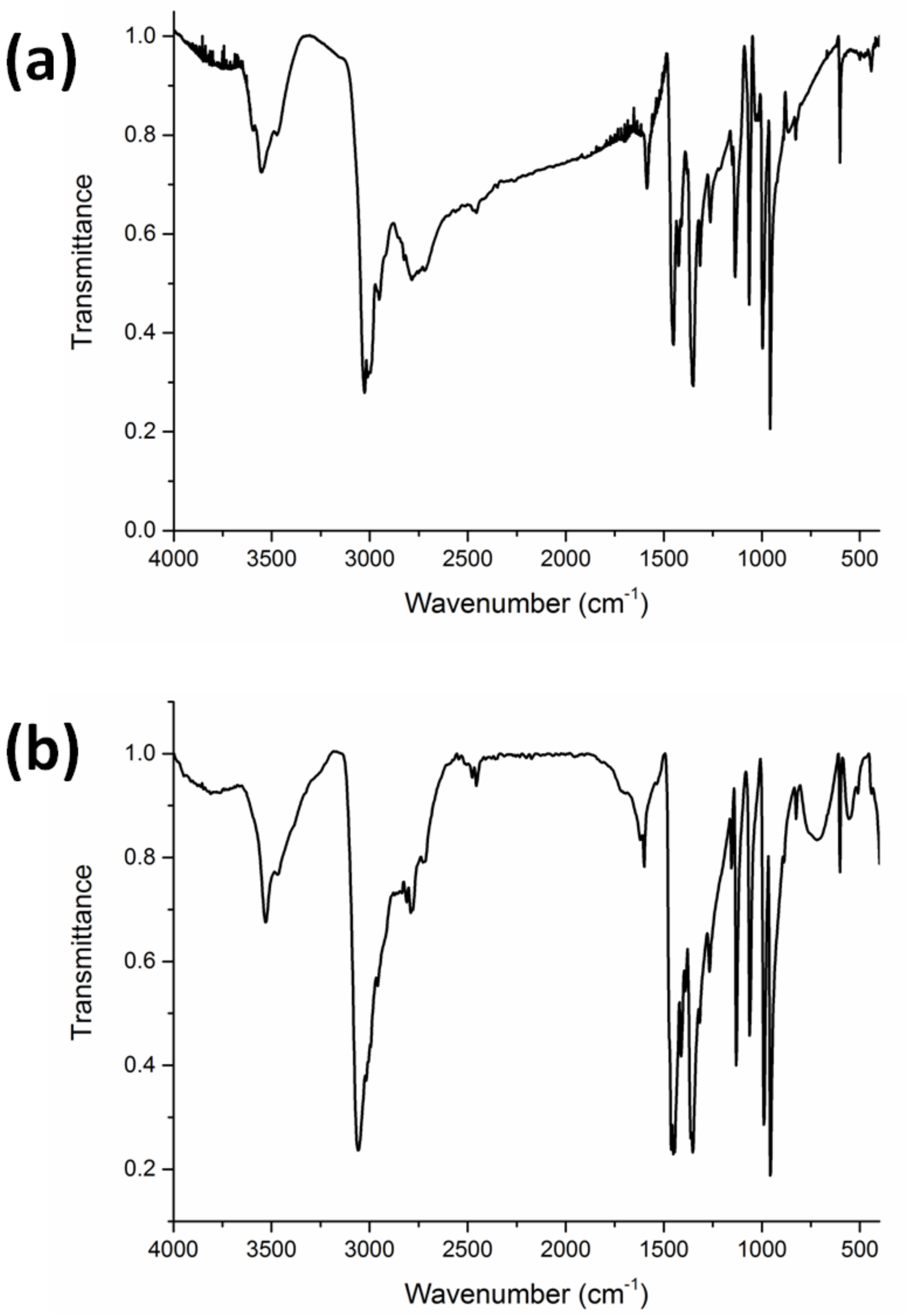


**Figure S2.** FTIR spectra of (a) compound 1, and (b) compound 2.

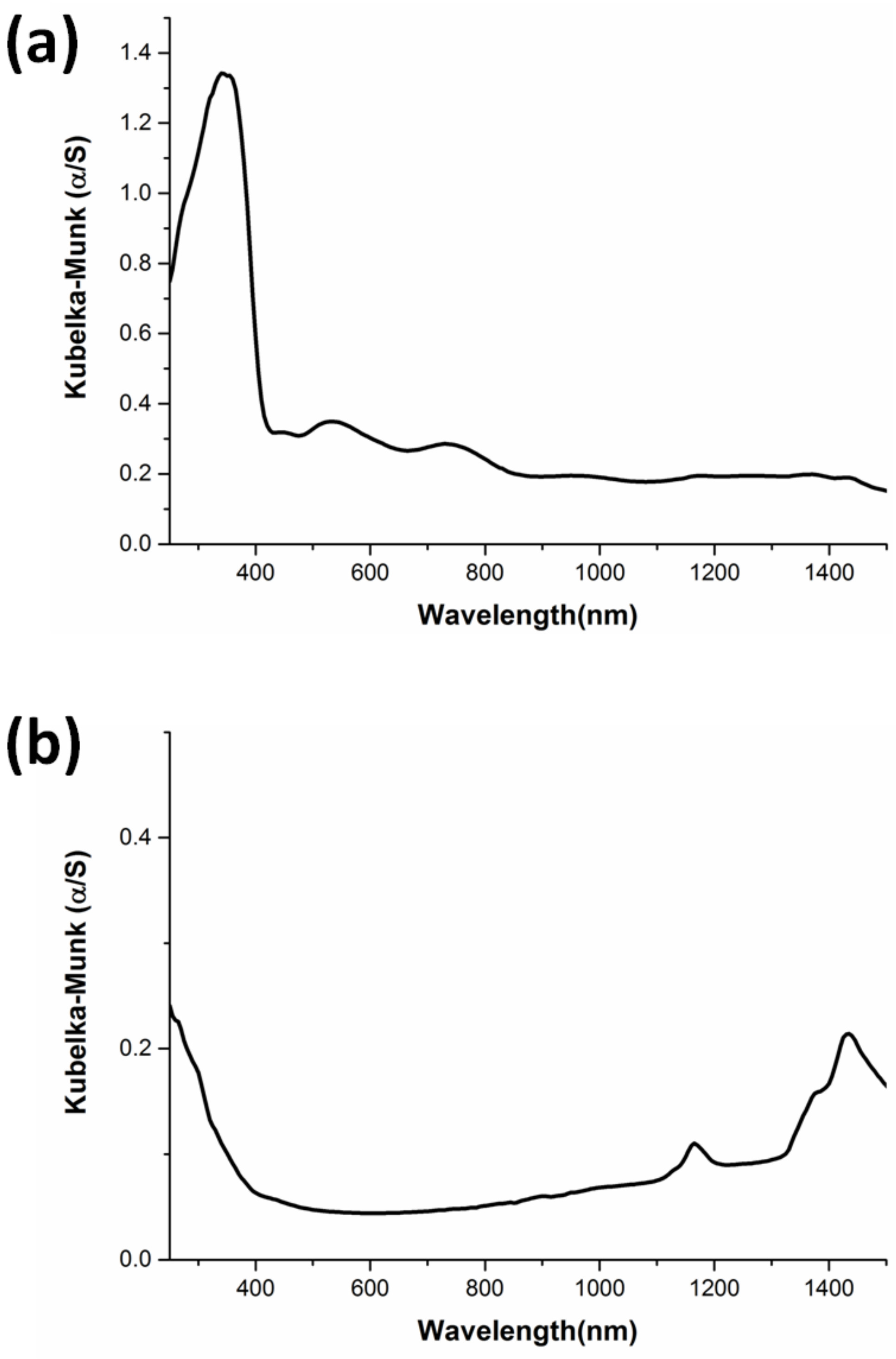


**Figure S3.** Absorption spectra for (a) compound 1, and (b) compound 2.

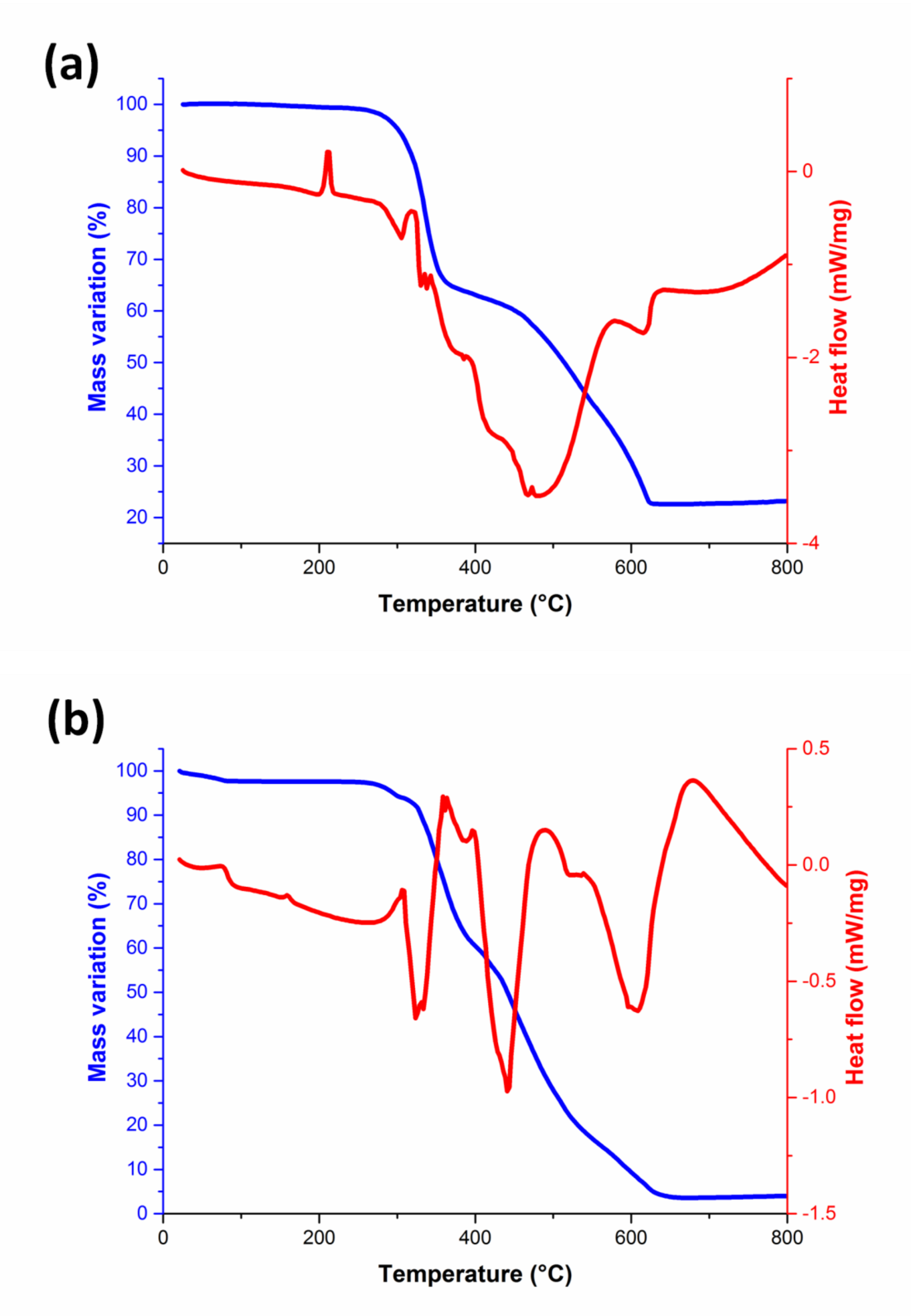


**Figure S4.** Thermogravimetric and DSC analyses for (a) compound 1, and (b) compound 2.

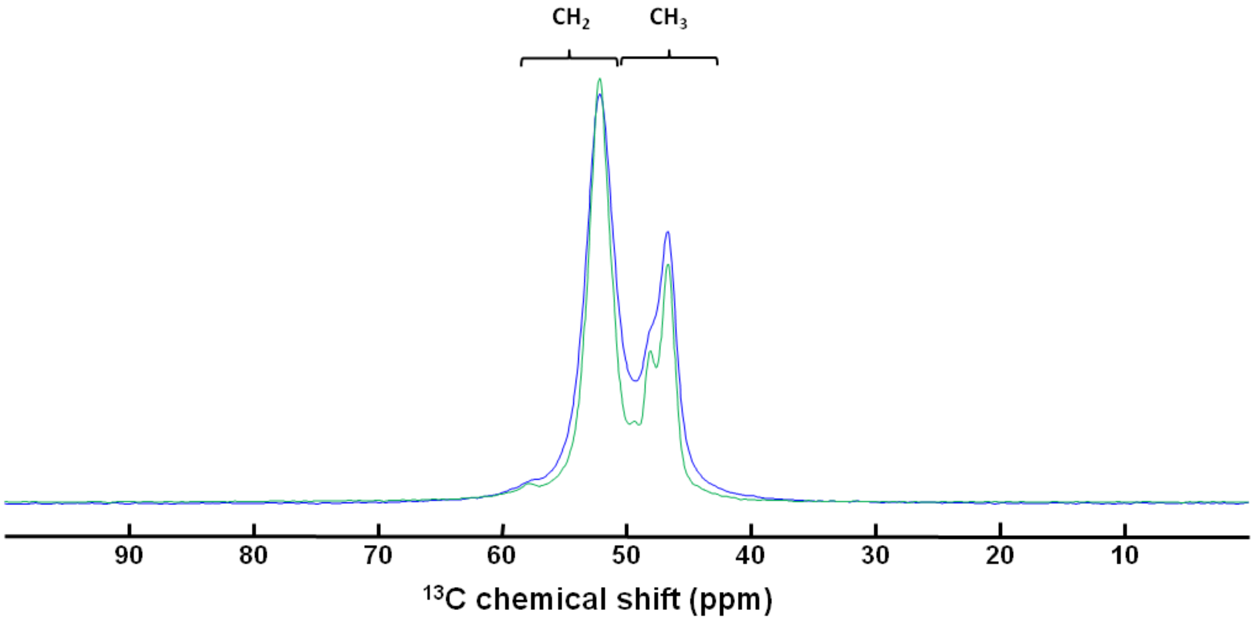


**Figure S5.** {$^{1}$H}-$^{13}$C CP-MAS NMR spectra at 300 MHz (blue) and 500MHz (green).

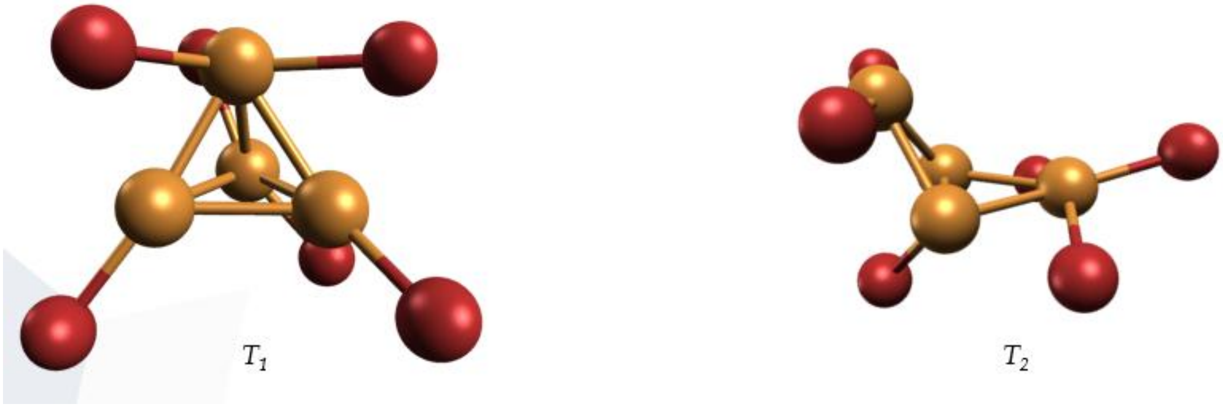


**Figure S6.** Illustration of the optimized geometries at the ES.

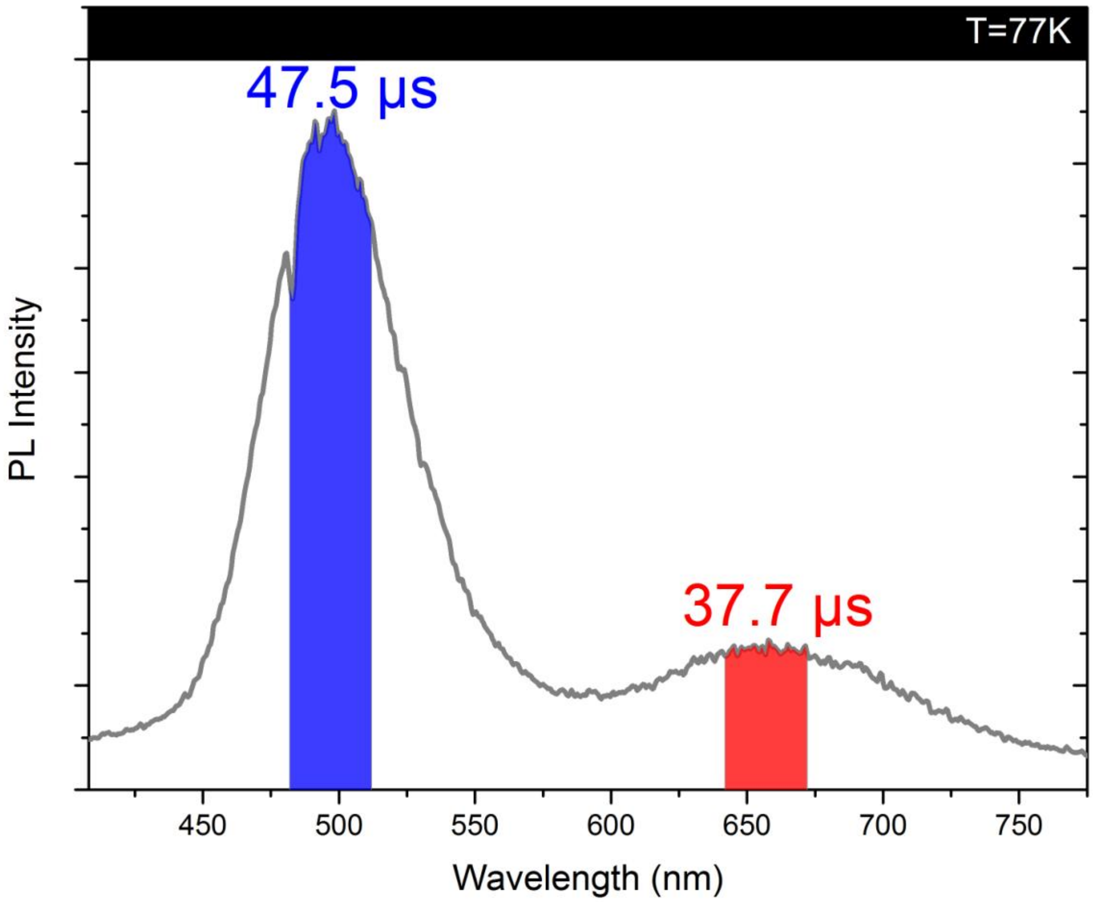


**Figure S7.** Time-resolved PL spectrum integrated over the entire 200µs window at 77K ($\lambda_{exc}$=267 nm).
